# Investigating Metabolic and Molecular Ecological Evolution of Opportunistic Pulmonary Fungal Coinfections: Protocol for a Laboratory-Based Cross-Sectional Study

**DOI:** 10.2196/48014

**Published:** 2023-08-15

**Authors:** Israel Kiiza Njovu, Pauline Petra Nalumaga, Lucas Ampaire, Edwin Nuwagira, James Mwesigye, Benson Musinguzi, Kennedy Kassaza, Kabanda Taseera, James Kiguli Mukasa, Joel Bazira, Jacob Stanley Iramiot, Andrew Baguma, Felix Bongomin, Richard Kwizera, Beatrice Achan, Michael J Cox, Jason S King, Robin May, Elizabeth R Ballou, Herbert Itabangi

**Affiliations:** 1 Medical Mycology Unit Department of Microbiology, Faculty of Medicine Mbarara University of Science and Technology Mbarara Uganda; 2 Department of Medical Laboratory Sciences Faculty of Medicine Mbarara University of Science and Technology Mbarara Uganda; 3 Department of Internal Medicine Faculty of Medicine Mbarara University of Science and Technology Mbarara Uganda; 4 Department of Medical Laboratory Science Faculty of Health Sciences Muni University Arua Uganda; 5 Department of Microbiology and Immunology School of Health Sciences Soroti University Soroti Uganda; 6 Mycology Unit Department of Microbiology and Immunology Busitema University Mbale Uganda; 7 Department of Microbiology School of Medicine Kabale University Kabale Uganda; 8 Department of Microbiology and Immunology Faculty of Medicine Gulu University Gulu Uganda; 9 Infectious Diseases Institute College of Health Sciences Makerere University Kampala Uganda; 10 Department of Microbiology School of Biomedical Sciences, College of Health Sciences Makerere University Kampala Uganda; 11 Institute of Microbiology and Infection College of Medical and Dental Sciences University of Birmingham Birmingham United Kingdom; 12 School of Biosciences Sheffield University Sheffield United Kingdom; 13 Medical Research Council Centre for Medical Mycology University of Exeter Exeter United Kingdom

**Keywords:** pulmonary mycoses, fungal-bacterial coinfection, metabolic, evolutionary, opportunistic infections, cross-kingdom interaction, tuberculosis

## Abstract

**Background:**

Fungal-bacterial cocolonization and coinfections pose an emerging challenge among patients suspected of having pulmonary tuberculosis (PTB); however, the underlying pathogenic mechanisms and microbiome interactions are poorly understood. Understanding how environmental microbes, such as fungi and bacteria, coevolve and develop traits to evade host immune responses and resist treatment is critical to controlling opportunistic pulmonary fungal coinfections. In this project, we propose to study the coexistence of fungal and bacterial microbial communities during chronic pulmonary diseases, with a keen interest in underpinning fungal etiological evolution and the predominating interactions that may exist between fungi and bacteria.

**Objective:**

This is a protocol for a study aimed at investigating the metabolic and molecular ecological evolution of opportunistic pulmonary fungal coinfections through determining and characterizing the burden, etiological profiles, microbial communities, and interactions established between fungi and bacteria as implicated among patients with presumptive PTB.

**Methods:**

This will be a laboratory-based cross-sectional study, with a sample size of 406 participants. From each participant, 2 sputa samples (one on-spot and one early morning) will be collected. These samples will then be analyzed for both fungal and bacterial etiology using conventional metabolic and molecular (intergenic transcribed spacer and 16S ribosomal DNA–based polymerase chain reaction) approaches. We will also attempt to design a genome-scale metabolic model for pulmonary microbial communities to analyze the composition of the entire microbiome (ie, fungi and bacteria) and investigate host-microbial interactions under different patient conditions. This analysis will be based on the interplays of genes (identified by metagenomics) and inferred from amplicon data and metabolites (identified by metabolomics) by analyzing the full data set and using specific computational tools. We will also collect baseline data, including demographic and clinical history, using a patient-reported questionnaire. Altogether, this approach will contribute to a diagnostic-based observational study. The primary outcome will be the overall fungal and bacterial diagnostic profile of the study participants. Other diagnostic factors associated with the etiological profile, such as incidence and prevalence, will also be analyzed using univariate and multivariate schemes. Odds ratios with 95% CIs will be presented with a statistical significance set at *P*<.05.

**Results:**

The study has been approved by the Mbarara University Research Ethic Committee (MUREC1/7-07/09/20) and the Uganda National Council of Science and Technology (HS1233ES). Following careful scrutiny, the protocol was designed to enable patient enrollment, which began in March 2022 at Mbarara University Teaching Hospital. Data collection is ongoing and is expected to be completed by August 2023, and manuscripts will be submitted for publication thereafter.

**Conclusions:**

Through this protocol, we will explore the metabolic and molecular ecological evolution of opportunistic pulmonary fungal coinfections among patients with presumptive PTB. Establishing key fungal-bacterial cross-kingdom synergistic relationships is crucial for instituting fungal bacterial coinfecting etiology.

**Trial Registration:**

ISRCTN Registry ISRCTN33572982; https://tinyurl.com/caa2nw69

**International Registered Report Identifier (IRRID):**

DERR1-10.2196/48014

## Introduction

### Overview

Fungi are ecologically regarded as environmental saprophytes that feed on decaying organic matter. However, over the past 30 years, fungi have transitioned into key etiological agents for difficult-to-manage infections, killing at least 1 million people each year. Yet they remain among the most neglected diseases worldwide [1,2]. The most serious invasive fungal infections often occur in the immunocompromised patients, such as those with HIV, cancer, and organ transplant, all of which have increased dramatically in the last 20 years [2]. Clinical manifestations of fungal infections have ranged from superficial to disseminated diseases with rates of invasive opportunistic fungal infections having upsurged during recent decades. Opportunistic pulmonary fungal coinfections are also emerging due to recent changing trends in predisposing factors and etiology. In Africa, pulmonary fungal coinfections are reported in about 15%-35% of cases, mainly within the cohort of individuals coinfected with HIV and tuberculosis (TB) [3,4]. This is posing enormous challenges to health care professionals, especially in resource-limited settings, where the diagnosis is not precise.

The chronic pulmonary disease has been linked with fungal etiology in the past but is often ignored. However, there is an emerging and interesting twist to fungal etiology, where cross-kingdom interactions appear to impact fungal virulence but are not fully explored. For instance, there are reports that bacteria can influence eukaryotic biology through various roles, such as parasites, commensals, or beneficial symbionts, in addition to acting as sources of new genetic sequences through subtle mechanisms like horizontal gene transfer [4-7]. In this context, the coexistence of fungi with other microbes, such as bacteria, in the same infection niche may unveil highly coordinated metabolic or molecular interaction profiles. This has been shown in chronic wound infections, where fungal-bacterial coexistence disrupts the highly controlled sequence of wound healing [3,8]. In fact, chronic wound microbiome and mycobiome studies reveal that fungal coinfections contribute to impaired wound healing [8]. A study by Mucunguzi and colleagues [9] reported a 22.2% prevalence of pulmonary fungal infections among people living with HIV in Southwestern Uganda. Therefore, it is possible that fungi also affect the outcome of chronic pulmonary diseases but are missed due to the lack of specific clinical or diagnostic signatures, leading to high rates of morbidity and mortality [3,10,11].

In the immunocompromised cohort, pulmonary infections can be exacerbated by a range of pulmonary opportunistic fungal coinfections. Unfortunately, infected individuals often present with symptoms similar to those of the most prevalent diseases, such as pulmonary tuberculosis (PTB) or bacterial pneumonia [3]. This propagates their rapid spread and augments poor prognosis. Hitherto, fungi co-isolated with bacteria have been considered irrelevant and often regarded as environmental contaminants [12]. This is further worsened by limited exposure of clinicians and laboratory fraternity to fungal infection biology. Nevertheless, patients could benefit from the definitive diagnosis of these infections and an exploration of the underlying mechanisms that drive saprophytic fungi to cause diseases in this patient population.

The changing trend of fungal etiology calls for the employment of advanced technology in profiling etiological agents. The synergistic ability of fungi to interact with other microbes, such as bacteria, in health and disease proves complex and can unveil new etiological agents and highlight novel virulence traits and antimicrobial targets. In this project, we propose to study the coexistence of fungal and bacterial microbial communities during chronic pulmonary disease, with a keen interest in underpinning fungal etiological evolution and the predominating interaction relationships that may exist between fungi and bacteria. There is already evidence that such interactions may culminate into properties distinct from those of their individual consortia [4,7,13-17]. Three hypotheses define this study, specifically that the following: (1) the burden of opportunistic pulmonary fungal coinfections is higher than what culture-dependent data suggest; (2) culture-independent data complement culture-dependent data in supporting therapeutic decisions; and (3) fungal-bacterial coexistence influences fungal and bacterial virulence. Thus, through these questions, we hope to reveal elusive interplays between the 2 microbial communities during disease development to explain differences in patient outcomes.

### Significance of the Study

Through this study, we can improve diagnosis and enhance clinical relevance as well as our epidemiological understanding of cross-kingdom etiological interactions. Pilot explorations of polymicrobial interactions, including bacterial-fungal interactions, can mediate a shift in substrate consumption, enzyme production, sexuality, thermostolerance, antimicrobial resistance, metabolism, and virulence [4,7,17-26]. For instance, some Mucorales fungi harbor bacterial endosymbionts that significantly modulate their metabolic profiles and virulence attributes in plants [16,17,27,28]; a similar interaction strategy is suggested in animal hosts [29,30].

During blight light disease of rice seedlings, a saprophytic fungus, *Rhizopus microsporus*, benefits from a toxicogenic alliance formed between this fungus and its endosymbiont bacteria, *Burkholderia rhizoxinica* or *Burkholderia endofungorum*; this alliance enables them to produce a potent anti–beta-tubulin toxin rhizoxin, which is a hepatotoxic cyclopeptide [17,27,31]. In addition, a range of phytotoxins with the potential to mediate virulence, which is thought to be produced by fungi, is a consequence of such interaction [16].

Although the impact of such relationships on clinical outcomes remains unclear, we recently demonstrated that another bacterial endosymbiont, *Ralstonia pickettii*, enabled *Rhizopus microsporus* to modulate innate immune responses in an animal model via a novel secreted factor [29,30]. Removing the endosymbiont using antibiotics rendered *Rhizopus microsporus* completely avirulent. These bioactive compounds are, therefore, important for fungal virulence as well as having potentially exploitable activities as demonstrated by the long history of fungal natural products in medicine, agriculture, and research [32].

### Study Aims

This study aims to determine and characterize the burden, etiological profiles, microbial communities, and interaction relationships established between fungi and bacteria implicated in pulmonary fungal coinfections.

## Methods

### Study Design

This study will be a laboratory-based observational cross-sectional study.

### Study Setting and Population

The study is being conducted at Mbarara Regional Referral and Teaching Hospital (MRRH) in Southwestern Uganda among patients suspected of having PTB.

### Selection Criteria

The target population under study consists of patients suspected of TB at MRRH. Participants will be included if they are 18 years and older, provide consent, and have at least one of the following qualities: presenting with PTB-like symptoms, having no prior history of TB, testing positive or negative on smear test and GeneXpert test, exhibiting lipoarabinomannan antigen positivity along with persistent respiratory symptoms, and being on anti-TB treatment and severely ill yet able to produce sputum. Conversely, HIV-positive or HIV-negative patients who have TB-like symptoms but are on anti-TB treatment with a favorable prognosis will also be included. Patients who are on anti-TB treatment and severely ill but unable to produce sputum, critically ill patients, and pregnant women will be excluded from the study. Patients who are critically ill are excluded because their condition may cause delays in sample collection and obtaining consent, which would impact the recruitment process. Additionally, pregnant women suspected of TB are excluded since they are managed differently according to TB guidelines.

### Statistical Analysis Plans and Power Calculations (Sample Size)

Based on the preliminary study, the prevalence of fungal coinfections among patients suspected of TB has been previously determined to be 40% using Kish-Leslie formula (n=Z^2^ P (1-P)/ d^2^) for determining the sample size in a cross-sectional study. In this formula, n is the sample size, Z (1.96) is the statistic for the level of confidence, P (0.4) is the estimated prevalence, and d (0.05) is the margin of error for the confidence interval. Using the Open Epi calculator, considering a population of 195,013 for Greater Mbarara, the sample size was determined to be 369 patients. Since we anticipate a 10% dropout rate, we will recruit 406 patients. From these patients, only 2 sputum samples will be collected—an on-spot sample on the day of recruitment and an early morning sample the following day, making a total of 812 samples to be collected. Obtained data will be analyzed using appropriate software, such as GraphPad (GraphPad Software Inc), Stata (StataCorp), or Epi Info (Centers of Disease Control and Prevention), among many others.

### Recruitment Strategy and Retention

Patients will be recruited by convenient sampling as they report for medical services at the HIV/TB clinic of MRRH. The recruitment of the participants will follow the existing diagnosis and point-of-care procedures at both study sites. The clinic receives an average of 10 new cases per day. Considering both our inclusion and exclusion criteria, we hope to recruit an average of 4 patients per day, resulting in an average total of 88 patients per month. This recruitment plan is achievable based on the rate of patients’ inflow at the clinic. Additionally, sputum sample collection is not an invasive method that would scare away patients, so we do not anticipate any major challenges with patient retention. However, we will factor in a possible 10% dropout rate among patients, and thus, the final sample size will be increased by 37 more patients, resulting in a total of 406 patients, as previously mentioned.

### Recruitment Site Selection

We have selected Uganda for this study because the country ranks high among the severely affected nations with HIV and TB pandemics. This is particularly true in western Uganda, where Mbarara (the proposed study site) is located. This subpopulation is also ranked among those most at risk of opportunistic fungal coinfections, yet it has been grossly ignored. Opportunistic pulmonary fungal coinfections are not uncommon among the immunosuppressed cohort, such as patients with HIV. We have selected patients suspected of having TB because most of the opportunistic pulmonary fungal infections present like TB, and therefore, are often missed during routine diagnosis. We have selected Mbarara Hospital as the study site because it is a regional referral hospital for the western region of Mbarara and a treatment center for patients with HIV or TB. Thus, we will have easy access to our study cases due to the high incidence of HIV or TB in this community. However, several population-based studies have been conducted in the past, focusing on HIV and TB, but are not associated with fungal coinfections [33,34]. Additionally, Mbarara University of Science and Technology has the infrastructure to handle most of the study demands. For instance, the Department of Microbiology in the Faculty of Medicine hosts a microbiology laboratory with medical mycology and TB units that have the capacity to respectively perform routine fungal or TB-based sputum analytical protocols, such as TB and GeneXpert testing, potassium hydroxide, and lactophenol cotton blue staining, as well as fungal and bacterial cultures. Additionally, the department has the capacity to do molecular-based procedures, including DNA isolation, polymerase chain reaction (PCR) amplification, and sequencing. The Department of Microbiology has also established collaborations with both national and international institutions, such as the Infectious Diseases Institute of Makerere University, Gulu University, Soroti University, and Muni University in Uganda; Massachusetts General Hospital and University of Minnesota in the United States; University of Birmingham, Sheffield University, and University of Exeter in the United Kingdom; and University of Aberdeen in Scotland.

### Approach and Methodology

#### Aim 1: Determining the Burden and Etiological Profiles of Fungal Coinfections Among Patients Suspected of TB

Patients' sputum samples will be collected and cultured on both fungal and bacterial media for either organism’s isolation. The isolates will be identified and profiled accordingly using conventional phenotypic and genomic-based assays. Total DNA will be extracted from the same sputum samples using a bead-beating phenol-chloroform approach. PCR primers targeting region 2 intergenic transcribed spacer (ITS2) will be used to amplify the fungal DNA present in the sputum samples; the amplified DNA will then be sequenced by Illumina MiSeq technology. This approach has been demonstrated to sensitively identify and determine the relative abundance of all fungi present in similar respiratory samples [35]. In this study, we will use this approach to identify the most abundant fungi present and detect any potential coinfections, which maybe challenging to identify using traditional culture methods. Fungal data will be analyzed using Mothur and the R statistical package Phyloseq (R Core Team) and will be speciated with reference to the UNITE2 database. Fungal quantitative PCR will be applied to determine the total burden of fungi present in each sample. Shotgun metagenomics will also be applied to a subset of samples. This metagenomics approach will reveal genomic detail of the fungi present, which will potentially include antimicrobial resistance genes and virulence genes and provide further typing information that reveals common clones or transmission among patients. Using long-read technology, the Oxford Nano pore GridION has the advantage of being a rapid sequencing technology that has shown promise as a rapid point-of-care test for the identification of microorganisms in clinical samples. As such, the use of this technology in this study may also reduce the time until diagnosis for fungal infections.

#### Aim 2: Establishing and Characterizing Microbial Communities Associated With Patients Suspected of Having TB

16S ribosomal RNA (rRNA) gene sequencing will be used to target the bacterial communities present in the same samples, using the same DNA extract, to identify bacterial coinfections. The results obtained will be used to describe the bacterial communities present in the sputum samples and then will be integrated with ITS2 fungal data [13,36-38]. To determine the association with the host phenotypes, the clinical metadata, including but not limited to age, gender, BMI, race or tribe, behaviors, and immune status, will be used to determine the association between fungal and bacterial communities with host phenotypes. For correlations between taxa, a combination of 16S rRNA gene and ITS2 sequencing data from the same samples will be used. Co-abundance patterns of bacteria and fungi will reveal potential interactions between and within fungal-bacterial communities, which can be further explored using DNA-independent methods in Aim 3. Both fungal-fungal, bacterial-bacterial and fungal-bacterial relationships will be interrogated using appropriate software packages, such as SparCC [38,39].

#### Aim 3: Characterizing Potential Interaction Relationships Established Between Fungal and Bacterial Communities

We will use a metabolomics approach in which sputum samples will be homogenized with methanol for metabolite extraction and derivatization. The extract and derivatives will then be analyzed for chemical composition using quantitative gas chromatography-mass spectrometry technique based on metabolite profiling [40]. Gas chromatography-mass spectrometry–based metabolite raw data will be processed using the XCMS toolbox; this processing will involve peak detection, alignment, and multivariate statistical analysis using the XCMS outputs.

We will also attempt to design a genome-scale metabolic model for pulmonary microbial communities to infer the entire microbiome (bacteria and fungi) and host-microbial interactions under different patient conditions. This will be based on the interplays of genes (identified by metagenomics and inferred from amplicon data) and metabolites (identified by metabolomics) by analyzing the full data set and using specific computational tools, including but not limited to Meta-genomic Operationalized Assembly and Annotation Tool 2 for functional categorization and Human Microbiome Project Unified Metabolic Analysis Network 2 for profiling abundance of microbes and activity of their metabolic pathways. A genome-scale model will be developed to explore complex relationships between microbial components, such as genes, using assembly of gut organisms through reconstruction and analysis, a reconstruction-based tool used for microbial-host metabolism, and interactions’ analysis [41,42]. In this manner, a genome-scale metabolic model will help us to postulate and test the underlined hypotheses that could link the burden, genotype, and phenotype of pulmonary microbial communities. Through these analyses, we also hope to infer ecological relationships, including but not limited to mutualism, commensalism, competition, antagonism, and symbiosis.

### Expected Deliverables

The following will be expected deliverables to be achieved during the project:

- Total fungal burden in 406 patients and its changes over time (using quantitative PCR)
- Fungal microbiome analysis: relative abundance of sputum fungi and their identity (using ITS2 sequencing)
- Bacterial microbiome analysis: relative abundance of sputum bacteria and their identity (using 16S rRNA gene sequencing)
- Whole community metagenomics: detailed genomic identification and characterization of abundant members of the sputum microbiome, including gene-level detail
-  Metabolomics profiling of sputum: analysis of host and microbial metabolites in longitudinal sputum samples
-  Integration of culture-dependent and culture-independent data with clinical information to understand disease processes in the patient cohort
- A genome-scale metabolic model of the disease
- Identification of fungal and bacteria in the disease to support subsequent epidemiological, treatment, and basic research investigations
- Training and knowledge transfer in cutting-edge microbiome techniques

### Study Safety

Study participants will be patients with infectious diseases and will be subjected to minimal risks during sample collection. However, the study will involve the collection, handling, and processing of contagious infectious human biological materials, such as sputum. Additionally, some of the proposed laboratory procedures will involve the use of hazardous reagents, which raise health and safety issues for both study staff and the general environment. However, to mitigate the anticipated risks, we will perform risk assessment procedures to identify the associated risks. Following risk assessment, we will develop standard operating procedures and comply with Control of Substances Hazardous to Health Regulations. These procedures will highlight health and safety measures conforming to the relevant local, national, or international guidelines. To this end, necessary precautions will be taken to ensure that these legislations are followed by both participants and staff involved in the metabolic and molecular ecological evolution of opportunistic pulmonary fungal coinfections (MeMoF) project.

### Study Registration

After a careful audit of the protocol and design, the study was registered in the International Standard Randomized Controlled Trial Number registry (ISRCTN33572982).

### Ethics Approval

The study has been approved by the Uganda National Council of Science and Technology (HS1233ES) and Mbarara University Research Ethics Committee (MUREC1/7-07/09/20) and will be conducted according to Uganda National and European Union legislation.

### Informed Consent

Target patients will be fully informed of the purpose and procedures of this study, their obligations, potential benefits, and risks. To this effect, an informed consent form was prepared and approved by the institutional review board of Mbarara university for use; the form will be signed voluntarily by each of the study participants prior to enrolment.

## Results

Study design, ethical review, approval, and research registration have all been completed. Patient enrollment commenced in March 2022. Most of the enrolled patients have completed their 2 visits. Patient enrollment was completed in June 2023, and the study results will be submitted for publication in late 2023.

We will share the study findings with the department of Microbiology, Faculty of Medicine, Mbarara University of Science and Technology; Ministry of Health Uganda; and other partner nongovernment organizations working with TB and related coinfections. We will discuss the scope, impact, and limitations of pulmonary fungal coinfections and bacterial fungal synergism based on our study findings. We hope to achieve technical and policy-level interventions, conduct capacity building, and increase public awareness of microbial ecological evolution. Finally, we will write abstracts and manuscripts for international conferences to an international audience and for peer-reviewed journals, respectively.

## Discussion

### Expected Findings

We anticipate that the findings of our study will enhance understanding and awareness of invasive pulmonary fungal coinfections among patients suspected of having TB. We hope that the diagnosis of invasive pulmonary fungal infections will improve, thereby advancing patient prognosis. In this study, we highlight an important aspect of cross-kingdom synergism during infection. In particular, we will highlight that fungal-bacterial coinfection is common among the susceptible cohorts of patients, particularly patients suspected of having TB. Our study aims to establish and characterize a network of microbial communities associated with chronic pulmonary diseases, in addition to TB. In doing so, we also hope to characterize potential interaction relationships established between fungal and bacterial coinfected communities.

### Previous Findings

PTB is the most common opportunistic pulmonary disease, especially among the cohort infected with HIV/AIDS [3,33]. However, the prevalence of TB has been masked by other possible and somewhat endemic infections that can be as common [43-45]. Accordingly, invasive pulmonary fungal coinfections have been highlighted. Several studies show that endemic pulmonary mycoses are not uncommon; however, clinically, many of them manifest with similar signs to TB [45]. This seems to have promoted the misdiagnosis of many pulmonary fungal infections. In other instances of fungal infections, such as aspergillosis, the treatment of PTB is further complicated with residual activation. Thus, in many parts of the world, pulmonary fungal infections continue to be missed or misdiagnosed.

The spectrum of fungal etiology has evolved over the years, expanding from the commonly isolated Candida species to encompass clinically rare but commonly environmental opportunists, such as *Cladosporium* spp*, Acremonium* spp*, Alternaria* spp*, Aspergillus* spp, and *Fusarium* spp, among others. This evolutionary event seems to be directly proportional to a recent dramatic increase in predisposing conditions, such as solid organ transplantation, prolonged chemotherapy, and hematological malignancies, which have more than doubled over the last 20 years.

Although the etiology of chronic pulmonary infections is often attributed to *Mycobacterium tuberculosis*, several data reveal that numerous unappreciated fungal opportunists and other non-TB bacterial pathogens can also coinfect or serve as the primary cause of the chronic pulmonary disease [43,45]. Our preliminary hospital data show that up to 92% of patients suspected of having PTB develop pulmonary fungal colonization. Previously, Njovu et al [35] determined a 70.7% prevalence for pulmonary fungal coexistence among patients suspected of having TB. However, the causal profile of disease by most of these cocolonizers has not been explored yet. The challenge is that current diagnostic approaches are not designed to routinely assess polymicrobial infections, and therefore, may fail to detect the presence of fungal-bacterial coinfections.

In this protocol, we hypothesized that cocolonization of a host by multiple microbial communities is influenced by the environmental frequency and antimicrobial resistance profiles of both commensal bacterial and fungal opportunists; furthermore, understanding the nature, diversity, and environmental origin of these communities will provide important insight into their pathological potential. Sadly, not much attention has been given to the possibility of cross-kingdom etiology linking TB and pulmonary mycoses. Polymicrobial infection of the pulmonary system is not uncommon. Importantly, how the different etiological entities influence each other is mostly unknown. However, stemming from the wider environment, we know that bacteria and fungi interact on several levels and can have various effects within each consortium [4,7,17-26]. Yet the impact of these effects is barely known.

Taken together, our data so far suggest that fungal-bacterial coexistence may influence their virulence attributes and antimicrobial resistance patterns, with relevance to patient outcomes. Therefore, in this project, we will investigate how these cross-kingdom synergistic interactions in the wider environment shape the evolutionary trajectories of fungi and bacteria, priming them for antimicrobial resistance and virulence in mammals. Importantly, the diversity of pulmonary infective fungi and bacteria and their complex population structures pose challenges to the analysis and interpretation of conventional data.

### Strengths and Limitations

The main strength of our study is that it is the first research project to integrate fungal-bacterial interactions at the interface of metabolic and molecular ecological evolution of fungal-bacterial etiology that is conducted using direct patient samples. Thus, the methodological setup has been designed to enhance awareness of fungal etiology and fungal-bacterial interactions among patients suspected of having TB. Through this study, we hope to improve the diagnosis and management of pulmonary coinfections. The downside of the study is purely technical; we anticipate a limitation in our methodological approach to study key co-interaction effects of the etiological agents at the metabolic level. This limitation might be associated with our lack of capacity to perform metabolomics experiments.

### Conclusions

We present the protocol of a laboratory-based cross-sectional study involving patients with TB-like symptoms. Through this study, we will explore the metabolic and molecular ecological evolution of invasive opportunistic pulmonary fungal coinfections. This will be achieved by determining the fungal burden, characterizing the associated microbial communities and exploring the potential interaction relationships established between fungal and bacterial etiologies. Through this study, we aim to create diagnostic profile schemes that can be used to infer patient prognostic outcomes in at-risk individuals. Establishing key fungal-bacterial cross-kingdom synergistic relationships is crucial for instituting fungal-bacterial coinfecting etiology.

## Appendix

Multimedia Appendix 1 Peer-review reports from the European & Developing Countries Clinical Trials Partnership (Netherlands).

## Abbreviations

ITS2: intergenic transcribed spacer 2
MeMoF: metabolic and molecular ecological evolution of opportunistic pulmonary fungal coinfections
MRRH: Mbarara Regional Referral and Teaching Hospital
PCR: polymerase chain reaction
PTB: pulmonary tuberculosis
rRNA: ribosomal RNA
TB: tuberculosis

## Data Availability

All the data sets generated during this study will be available from the corresponding author as governed by the data-sharing act of Mbarara University of Science and Technology. All generated data sets will be deposited in publically available repositories or published as study manuscripts.
