# Supplementary material for: Investigating Metabolic and Molecular Ecological Evolution of Opportunistic Pulmonary Fungal Coinfections: Protocol for a Laboratory-Based Cross-Sectional Study
Source: JMIR Res Protoc. 2023 Aug 15;12:e48014. doi: 10.2196/48014 (PMC10466149; doi:10.2196/48014)
Supplement: Multimedia Appendix 1 [file resprot_v12i1e48014_app1.pdf]

EDCTP

European & Developing Countries Clinical Trials Partnership

**Reference**	**TMA2019CDF-2789**
**Title**	**Metabolic and molecular ecological evolution of opportunistic pulmonary fungal co-infections**
**Lead Applicant**	**Dr Herbert Itabangi**
**Organisation**	**Mbarara University of Science and Technology (MUST)**
**Reviewer**	**5**

Assessment - Rapporteur

Review

Excellence

Excellence - Review Comments
Overall the proposed project was found to be of high quality and falling within the scope of the EDCTP2 call, focusing on fungal co-infections which is an important and timely area of research. The concept, research questions and hypotheses are well defined, clear, and achievable. The project investigates important questions and has a strong likelihood of advancing the field. Aspects of the methodology, especially surrounding the clinical aspects, such as inclusion criteria and potential sources of bias, and sampling aspects such as the risk of contamination of pulmonary samples by the nasopharyngeal microbiome were identified as somewhat unclear and in need of strengthening. This could be done by identifying and strengthening the clinical support for the fellow. Outside of some aspects of the clinical work, the proposed methodology was thought to be generally appropriate. The expectations for capacity development of the fellow are high with clear development potential and fits well with the candidate's expertise and career plans. The mentorship and capacity development plans were noted as lacking in detail and could be further strengthened and detailed.<br><br>The candidate appears excellently suited to perform this research, having obtained his postgraduate research qualifications with leading mycologists in the UK, and currently heads the medical mycology unit in his institution. The candidate is also uniquely placed to further work in this area in his country, demonstrates excellent potential and independence and a vision for his research, and it appeared clear that this fellowship would advance his career and assist with establishing his research in his home institution.<br><br>The major concern raised by the committee was that ethical considerations have not been managed and the ethics self-assessment was not provided.<br><br>This is a major shortcoming which needs to be corrected.

Excellence - Score	4

Impact

Impact - Review comments
Overall, this project was expected to have high impact, across all measures of impact and with the scope of impact extending beyond the local context. The proposed work was also found to have a high expected impact both in terms of scientific/clinical advance and in potential for policy level input with the possibility of improving individual and population health. There is also clear evidence that individual and institutional capacity strengthening will result due to this project. There is a strong sense from the application that this fellowship will not only assist in training and helping establish this promising candidate as a leading researcher in mycology, but that there will be a far larger impact, on the country and the research community, the latter through increasing the pool of skilled workers, establishing collaborations with leading fungal labs in the UK and US, and strengthening the institution considerably. Plans to disseminate findings are appropriate and well described, including consideration of IP. Sustainability and retention of capacity beyond the end of the grant are high, with the fellow having a long-term post. Minor concerns were raised regarding some missing detail in terms of clinical data management, but none requiring significant changes.

**Impact - Score**	4.5

Quality and Efficiency of Implementation

**Implementation - Review comments**
Overall, the planning and implementation of the proposed work was very good. The work plan, scientific and training objectives, and resource allocation are all clear and well-aligned. Deliverables and milestones are clear and appropriate for the proposed work. The support offered by the host institution, as well as the specific nature of support by consortium members is excellent and there is high confidence that sufficient capacity exists to carry out the stated aims. Minor concerns were raised relating to the lack of letters of support or similar from the collaborating centres. Compliance with international standards of the researcher is clear, but some components are missing (as already noted, especially the ethics self-assessment). The candidate's permanent position at the hospital is underwritten, allowing the impact of this investment in his training to be continued into the future, as evidenced by a strong commitment from the mentor. Minor queries were raised at the lack of detail in some aspects of the implementation plan, including in risk management and in management structure, but none requiring substantive change.

**Implementation - Score**	4

**Total Score**	12.5
